# Deep Flow Cytometry Unveils Distinct Immune Cell Subsets in Inducible T Cell Co-Stimulator Ligand (ICOSL)- and ICOS-Knockout Mice during Experimental Autoimmune Encephalomyelitis

**DOI:** 10.3390/ijms25052509

**Published:** 2024-02-21

**Authors:** Davide Raineri, Hugo Abreu, Beatrice Vilardo, Natasa Kustrimovic, Chiara Venegoni, Giuseppe Cappellano, Annalisa Chiocchetti

**Affiliations:** 1 Department of Health Sciences, Interdisciplinary Research Center of Autoimmune Diseases-IRCAD, University of Eastern Piedmont, 28100 Novara, Italy; davide.raineri@uniupo.it (D.R.); hugo.abreu@uniupo.it (H.A.); beatrice.vilardo@uniupo.it (B.V.); natasa.kustrimovic@uniupo.it (N.K.); chiara.venegoni@uniupo.it (C.V.); annalisa.chiocchetti@med.uniupo.it (A.C.); 2 Center for Translational Research on Autoimmune and Allergic Disease-CAAD, University of Eastern Piedmont, 28100 Novara, Italy

**Keywords:** ICOSL, experimental autoimmune encephalomyelitis, memory cells, regulatory T cells, multiple sclerosis

## Abstract

The inducible T cell co-stimulator ligand (ICOSL), expressed by antigen presenting cells, binds to the inducible T cell co-stimulator (ICOS) on activated T cells. Improper function of the ICOS/ICOSL pathway has been implicated in several autoimmune diseases, including multiple sclerosis (MS). Previous studies showed that ICOS-knockout (KO) mice exhibit severe experimental autoimmune encephalomyelitis (EAE), the animal model of MS, but data on ICOSL deficiency are not available. In our study, we explored the impact of both ICOS and ICOSL deficiencies on MOG_35-55_ -induced EAE and its associated immune cell dynamics by employing ICOSL-KO and ICOS-KO mice with a C57BL/6J background. During EAE resolution, MOG-driven cytokine levels and the immunophenotype of splenocytes were evaluated by ELISA and multiparametric flow cytometry, respectively. We found that both KO mice exhibited an overlapping and more severe EAE compared to C57BL/6J mice, corroborated by a reduction in memory/regulatory T cell subsets and interleukin (IL-)17 levels. It is noteworthy that an unsupervised analysis showed that ICOSL deficiency modifies the immune response in an original way, by affecting T central and effector memory (T_CM,_ T_EM_), long-lived CD4^+^ T_EM_ cells, and macrophages, compared to ICOS-KO and C57BL/6J mice, suggesting a role for other binding partners to ICOSL in EAE development, which deserves further study.

## 1. Introduction

The inducible T cell co-stimulator (ICOS) molecule is a homodimeric type-I transmembrane receptor featuring an extracellular (Ig)V-like domain (characteristic shared with B7 family receptors) [1]. ICOS is primarily found on activated T cells but can be expressed on dendritic cells (DCs) as well [2]. Its unique binding partner is the inducible T cell co-stimulator ligand (ICOSL), a B7 homologous protein that is constitutively expressed on professional antigen presenting cells (APCs) and, to a lesser degree, on some other cell types, like endothelial cells (ECs), osteoclasts, and several tumor cell lines [3]. The interaction between ICOS and ICOSL plays a crucial role in governing T-cell activation within lymphoid organs and overseeing T-cell functionality at sites of inflammation. This has been extensively explored by utilizing knockout (KO) mice and stimulating or blocking molecules [4,5,6]. ICOS/ICOSL interactions also have the potential to shape a distinct microenvironment, exerting an impact on various cellular type differentiation, such as regulatory T cells (Tregs) or effector T cells, including T helper (Th)-17, follicular Th cells (Tfh), and the development of germinal centers. It is noteworthy that ICOS/ICOSL interactions trigger a process called reverse signaling, also influencing APCs. Indeed, this interaction modulates cytokine secretion, inhibits the adhesiveness and migration of DCs, ECs, and tumor cells, and inhibits osteoclast function [7,8,9,10]. This intricate crosstalk between ICOS/ICOSL becomes even more complex when considering recent findings from our laboratory and others. This evidences shows that ICOSL can also bind to osteopontin (OPN) [11] or other partners, such as ανβ_3_ integrin [12]. OPN is a matricellular protein that regulates the immune response at multiple levels [11,13], while ανβ_3_ is a member of the integrin superfamily of adhesion molecules involved in signal transduction and cell-to-cell interactions [14].

Multiple sclerosis (MS) is an autoimmune disease that primarily affects the central nervous system (CNS) and it is characterized by inflammation, demyelination, and axonal damage [15]. Co-stimulatory pathways facilitate the activation of T cells that infiltrate the CNS, significantly contributing to MS pathogenesis [16]. Activated CD4^+^ T cells with reactivity to myelin components have been identified in the blood and cerebrospinal fluid of individuals with MS. Furthermore, in acute and chronic MS lesions, CD4^+^ and CD8^+^ T cells were observed, respectively [17,18]. The most intensively studied animal model of MS is experimental autoimmune encephalomyelitis (EAE), due to its similarities to human disease in terms of both histopathology and immunological features. EAE is mainly mediated by helper CD4^+^ T cells that, following the activation in the periphery, enter CNS, where they trigger the activation of the resident microglia.

Dysregulation of the ICOS/ICOSL system has been implicated in several autoimmune diseases, including MS [2,19,20,21,22]. ICOS-KO mice develop an exacerbated form of chronic EAE but are relatively resistant to other autoimmune diseases, such as experimental autoimmune myasthenia gravis and collagen-induced arthritis [23,24]. The effect of ICOS deficiency on EAE has been investigated in both chronic [4,25] and relapsing–remitting EAE models [5]. Moreover, several studies have suggested the possibility that interfering with the ICOS/ICOSL binding can lead to potential modulation of the autoimmune response in EAE. In EAE induced by the transfer of CD4^+^ memory cells, inhibiting the ICOS/ICOSL interaction has a beneficial effect, whereas in EAE induced by the transfer of CD4^+^ effector cells, blockade of the ICOS/ICOSL pathway results in further deterioration [26]. Even though a substantial number of studies have confirmed the exacerbation of EAE in the case of a blockade or deficiency of ICOS, there are no data regarding the effect of ICOSL deficiency on EAE course.

Our study aimed to compare both ICOS and ICOSL deficiencies on EAE course and in the shaping of immunological response during the resolution phase of EAE. We thus induced EAE in ICOSL-KO, ICOS-KO, and C57BL/6J mice, followed the disease course until remission, and then performed a deep flow cytometry evaluation of splenocytes, which was subsequently analyzed by both classic and unsupervised analyses pipelines [27]. The enhanced capabilities of modern flow cytometers enable the utilization of multiparametric flow cytometry (up to 30 markers in the same tube), along with an unbiased analysis, that simultaneously incorporates all included markers to assess cell similarity. Clustering, as an unsupervised learning technique, categorizes unlabeled instances into meaningful groups based on their shared characteristics. The advantage of employing unsupervised clustering analysis lies in its ability to partition data in a more refined manner, thereby facilitating the discovery of novel and unexpected cell populations that may hold significance in the context of a specific disease.

The obtained results showed that ICOSL deficiency worsens the course of disease compared to C57BL/6J mice, paralleling that of ICOS-KO mice. Interestingly, ICOSL deficiency modifies the immune response in an original way, by affecting T central and effector memory (T_CM_, T_EM_), the long-lived CD4^+^ T_EM_ cells, and macrophages, compared to ICOS-KO and C57BL/6J mice. These results suggest that the ICOSL signaling pathway may contribute to their maintenance through a mechanism independent of ICOS.

## 2. Results

### 2.1. ICOSL Deficiency Exacerbates EAE and Impacts Recovery during Remission

**Figure 1:**
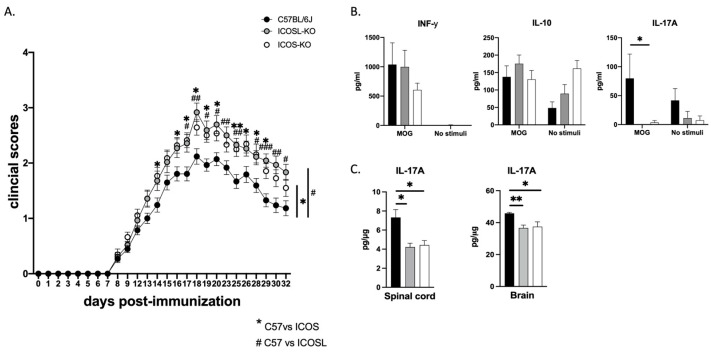
Clinical disease course of MOG_35-55_-induced EAE in C57BL/6J, ICOSL-KO and ICOS-KO mice. (**A**) EAE was induced using the established protocol in the laboratory (31). Results from three independent experiments are shown. Data are shown as mean ± SEM (n = 2830 mice/group). (**B**) Antigen-recall assay to MOG of splenic cells isolated from the three groups (n = 4–5) and cultured for 72 h in the presence/absence (no stimuli) of MOG_35-55_ peptide (10 μg/mL). (**C**) IL-17A protein levels (pg/µg) in brain and spinal cord homogenates from C57BL/6J, ICOSL-KO and ICOSK-KO mice. Data are shown as mean ± SEM (n = 5 mice/group). For statistical analysis, Kruskal–Wallis test with Dunnett multiple comparisons was used as a post-hoc test. The asterisks (*) or pound signs (#) depicted in the vertical line correspond to the statistical significance of the average daily disease score, which was computed from cumulative data across experiments. * *p* < 0.05; ** *p* < 0.01 C57BL/6J vs. ICOS-KO; # *p* < 0.05; ## *p* < 0.01; ### *p* < 0.001 C57BL/6J vs. ICOSL-KO.

In order to investigate the impact of ICOSL deficiency on EAE course, we induced chronic EAE in ICOSL-KO and compared the results with both C57BL/6J and ICOS-KO mice. EAE was significantly more severe in both KO mice in comparison with C57BL/6J mice, but no differences were found in the average disease scores, calculated using the cumulative data, between ICOS-KO and ICOSL-KO mice (Figure 1A).

During the remission phase, at the endpoint of the EAE experiment, we investigated whether the absence of either ICOS or ICOSL molecules would affect the production of MOG-driven specific cytokines. To achieve this, we performed an MOG-specific recall response of splenocytes harvested from both KO mice at the remission phase of the disease. No discernible differences regarding IFN-γ, IL-10, and IL-17A levels in both KO mice were found in the supernatant of MOG-stimulated splenocytes. However, the IL-17A cytokine was decreased in both KO mice when compared with C57BL/6J mice (Figure 1B). To rule out the possibility that this reduction reflected a real decrease in IL-17A-secreting cells in the spleen or was a consequence of their persistence in the brain and/or spinal cord, we examined IL-17A levels in the aforementioned target tissues. Interestingly, IL-17A protein expression was also significantly reduced in both the brains and spinal cords of ICOS-KO and ICOSL-KO mice (Figure 1C).

### 2.2. Deep Multiparametric Flow Cytometry Analysis with Classical Gating Strategy Reveals a Reduction in CD8^+^ T_CM_ Cells and Tregs and an Increase in CD4^+^ T_EM_ and Myeloid Cells in ICOSL-KO Mice

Since both KO mice cohorts exerted the same degree of disease severity, which could not be ascribed to differences in the cytokine production, we performed multiparametric flow cytometry analysis. The aim was to assess if the severity of EAE can be attributed to a specific immune cell subset.

We observed intriguing changes in the absolute numbers of various immune cell populations in both KO cohorts when compared to C57BL/6J mice. Notably, the ICOSL-KO cohort exhibited a significant increase in CD4^+^ T_EM_ compared to ICOS-KO; a similar trend was observed in C57BL/6J mice compared to ICOS-KO mice. We also found a downregulation in Tregs number and a trend in their activation status (detected through the upregulation of Helios marker, *p* = 0.067) in comparison with C57BL/6J mice [28].

**Figure 2:**
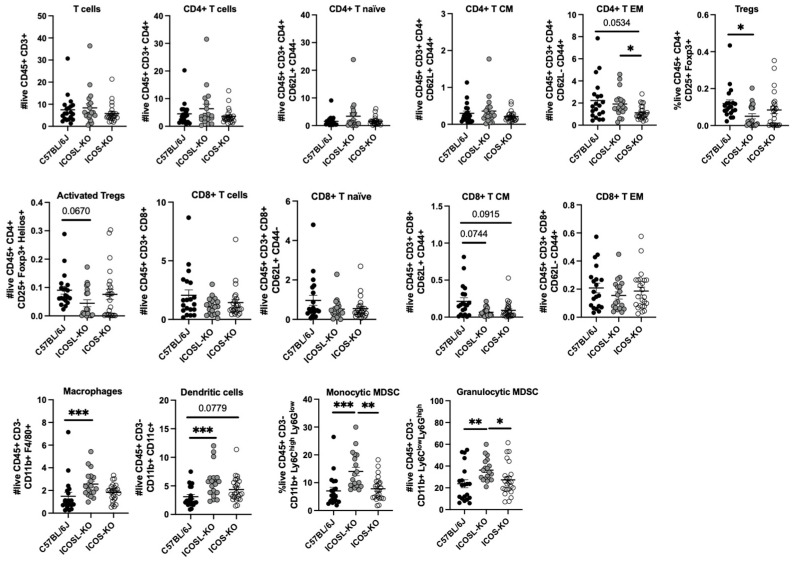
Multiparametric-flow-cytometry-supervised analysis of splenocytes from MOG_35-55_-induced EAE C57BL/6J, ICOSL-KO, and ICOS-KO mice. Each dot plot shows the absolute number (expressed as a power of 10^6^) of immune cell populations in the spleen (day 32 post-immunization) harvested from C57BL/6J, ICOSL-KO, and ICOS-KO mice. The samples were acquired using FACSymphony^TM^ A5 (Becton and Dickinson, San José, CA, USA) flow cytometer and data were analyzed using FACSDIVA software (Version 9.1, Becton and Dickinson, San José, CA, USA). CM: central memory; EM: effector memory; Tregs: regulatory T cells; MDSC: myeloid-derived suppressor cells. Data are shown as mean ± SEM of three independent experiments (n = 19–24 mice/group). For statistical analysis, Kruskal–Wallis test with Dunnett multiple comparisons as post-hoc test was used, * *p* < 0.05, ** *p* < 0.01, *** *p* < 0.001.

Regarding the CD8 compartment, both KOs showed a trend in the decrease in CD8^+^ T_CM_ in comparison with C57BL/6J mice (Figure 2).

Finally, ICOSL-KO mice showed an increase in the absolute number of macrophages and DCs in comparison with C57BL/6J mice. Monocytic and granulocytic MDSC absolute counts were also significantly increased in comparison with C57BL/6J and ICOS-KO mice (Figure 2).

### 2.3. Deep Flow Cytometry Unsupervised Analysis Reveals Distinct Cell Subsets and New Clusters Differentially Associated with ICOSL-Driven Disease

To investigate whether specific cell subsets were associated with ICOSL-driven EAE, we performed an unsupervised flow cytometry analysis. The unsupervised approach in flow cytometry is motivated by the need to explore and analyze complex datasets without predefined labels or classifications. In unsupervised methods, the algorithm identifies patterns, structures, or clusters within the data on its own, without relying on prior information/classification. This is particularly beneficial when dealing with heterogeneous samples or when the nature of the data is not well understood.

**Figure 3:**
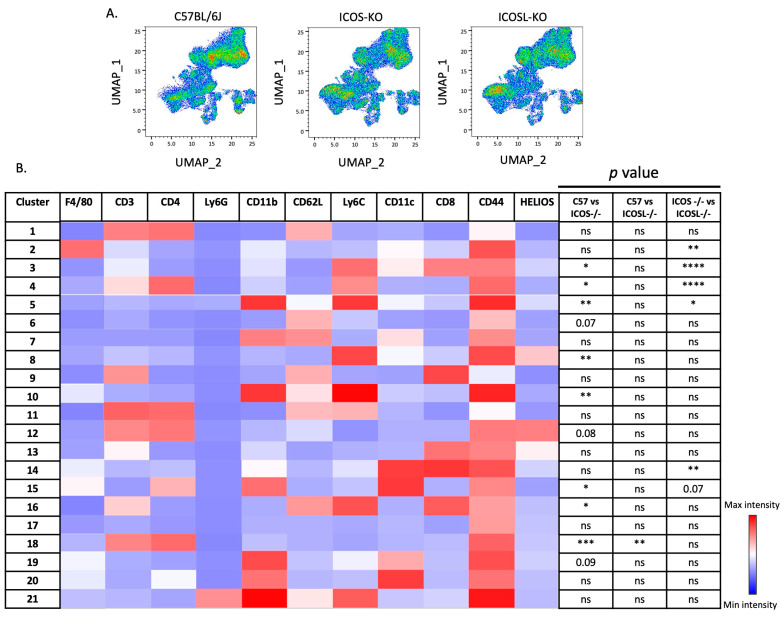
Unsupervised flow cytometry analysis identified 21 cell clusters. (**A**) UMAP multidimensionality reduction for C57BL/6J (**left**), ICOS-KO (**center**) and ICOSL-KO (**right**), concatenated and analyzed simultaneously. Data are shown as density dot plots. (**B**) Heatmap derived from the total median fluorescence intensity (MFI) (column-scaled z-scores) and comparison of the clusters among the three groups (C57BL/6J, ICOS-KO and ICOSL-KO). For statistical analysis, Kruskal–Wallis test with Dunnett multiple comparisons was used, ns = not significant, * *p* < 0.05, ** *p* < 0.01, *** *p* < 0.001 and **** *p* < 0.0001.

The UMAP algorithm was used to reduce the multidimensionality of the flow cytometry panel (Figure 3A) [29]. Subsequently, unsupervised clustering analysis employing the X-shift algorithm identified 21 clusters in both KO mice (Figure 3B) [30]. The latter algorithm efficiently analyzes datasets by rapidly estimating cell event density through the k-nearest-neighbor methodology, categorizing populations based on marker-defined classifications. This approach facilitates the automated clustering of cell subsets, revealing biological insights that might remain undiscovered when relying solely on “prior knowledge”.

**Figure 4:**
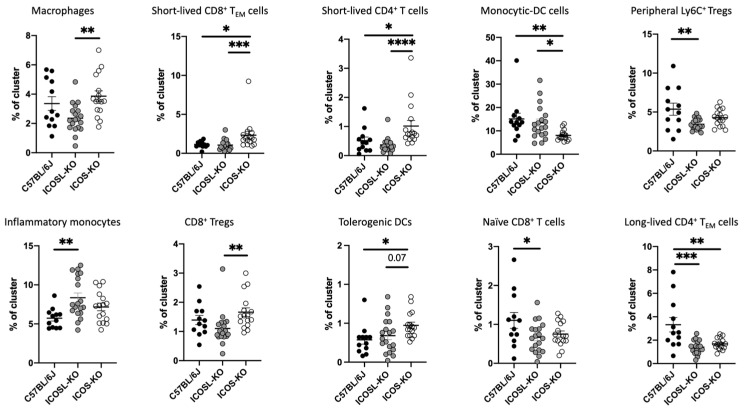
Unsupervised analysis reveals new cell subsets. Each dot plot shows the percentages of the depicted cells in C57BL/6J, ICOSL-KO, and ICOS-KO mice. For statistical analysis, Kruskal–Wallis test with Dunnett multiple comparisons was used. * *p* < 0.05, ** *p* < 0.01, *** *p* < 0.001, **** *p* < 0.0001. Data are shown as mean ± SEM of three independent experiments (n = 19–24 mice/group).

Ten out of the twenty-one clusters were significantly expressed at different levels among the genotypes, including C57BL/6J, as reported below (Figure 4).

To enhance clarity and facilitate understanding, the basic phenotypes and affiliations of the defined clusters are summarized in Table 1.

Unsupervised analysis revealed that the absence of either ICOS or ICOSL leads to some very specific alterations in the diverse cell populations that were not recorded by the conventional gating strategy analysis. The absence of ICOS leads to an increase in the pool of short-lived effector cells, both CD4 and CD8 (clusters 3 and 4, respectively), while the long-lived effector memory of CD4^+^ T cells is significantly impaired (decreased in cluster 18) in comparison to wt mice. Additionally, ICOS-KO exhibited an increase in tolerogenic-DCs (cluster 15).

Interestingly, the findings described above revealed differences between ICOS- and ICOSL-deficient mice in shaping the immune system, particularly in the percentages of macrophages, short-lived CD8^+^ T_EM_, short-lived CD4^+^ T cells, and CD8^+^ Tregs, with significant increases observed in ICOS-KO. These data may suggest that the ICOSL signaling pathway may contribute to their maintenance through a mechanism independent of ICOS.

## 3. Discussion

In this study, our objective was to explore the impact of both ICOS and ICOSL deficiencies on EAE (MOG_35-55-_induced) and its associated immune cell dynamics, employing ICOSL-KO and ICOS-KO mice, compared with C57BL/6J wtmice, which served as the control group.

Our results showed that both deficiencies are associated with the following: (i) the exacerbation of EAE compared to C57BL/6J mice and an overlapping EAE course; (ii) a significantly reduced recovery rate compared to C57BL/6J mice; (iii) a partially different phenotype of immune cells compared to C57BL/6J mice.

The interplay between costimulatory signals, such as ICOS/ICOSL, plays a key role in regulating T-cell activation and is believed to have a decisive influence in inciting and perpetuating cellular effector mechanisms in autoimmune diseases, such as MS.

Based on these observations, one could expect a milder clinical presentation in the case of ICOS-KO and ICOSL-KO mice compared to wt mice. However, Rottman et al. were among the first to challenge and dispel this assumption. The authors used a proteolipid protein (PLP) relapsing–remitting EAE model in which they blocked ICOS in wt mice by using ICOS-Ig, a recombinant molecule that binds to ICOSL on APCs, and thus impeded its binding to ICOS expressed on T cells. Surprisingly, this led to a different clinical course depending on the timing of the ICOS-Ig injection: during the efferent immune response (9–20 days after immunization), it led to the abrogation of EAE, while during the antigen priming phase (1–10 days after immunization), it led to exacerbation of the disease [5]. The results obtained in our study showed a similar exacerbation of EAE in ICOS-KO and ICOSL-KO mice when compared to C57BL/6J mice, thus highlighting the undoubted importance of the ICOS/ICOSL interaction in protecting against the development and progression of EAE.

ICOS is found on both CD4^+^ and CD8^+^ T cells, but there is a notable difference in ICOS mRNA expression between polarized Th2 and Th1 cells, with it being increased in polarized Th2 cells [40]. Furthermore, ICOS triggering induces IL-10 expression [41], and it has been shown that T cells from ICOS-deficient mice show deficiencies in IL-4 production [42,43]. Collectively, these data suggest that blockade of the ICOS–ICOSL pathway during antigen priming, either by the genetic deletion of ICOS or by treatment with a specific mAb to ICOS, results in polarization towards a Th1 response, thus offering a potential explanation for the exacerbation of the disease in ICOS-KO animals. Specifically, the elimination of ICOS from the overall scenario leads to a slowdown in the activation of anti-inflammatory Th2 cells, while the impact on the activation of pro-inflammatory Th1 cells is considerably less pronounced, resulting in an increased ratio of Th1:Th2 cells and leading to the exacerbation of symptoms in EAE, a prototype of Th1-mediated disease.

The culprit players in the development of EAE are activated Th1 and Th17 cells. ICOS is required for the differentiation of Th17 cells that secrete IL-17 [44] and the differentiation of Th1 cells, which are responsible for secretion of IFN-γ [45].

In our investigation, we noticed that MOG-restimulated splenocytes from both ICOS-KO and ICOSL-KO mice displayed reduced expression levels of IL-17A, with no discernible differences in the production of INF-γ. The unchanged production of INF-γ in ICOS-KO mice aligns with previous reports [1]. Regarding the production of IL-17A, the available literature presents contrasting results, with some studies asserting that the production of IL-17A is reduced [46], while others reported an increase in IL-17A production using the splenocytes of ICOS-KO animals [4]. Our data align with the data consistently emphasizing the importance of ICOS and ICOSL molecules in the differentiation and maintenance of Th17 cells [44,46].

In order to gain a more comprehensive understanding of the pathogenetic mechanisms underlying ICOS/ICOSL interactions in EAE pathology, we conducted an in-depth characterization of the differential immune responses through the use of deep immunophenotyping via multiparametric flow cytometry. The generated data were subject to analysis using both classical gating strategy (supervised) and unsupervised (multidimensionality reduction and clustering) approaches. This was carried out to achieve two main objectives: (i) gain an overview of the immune response, with a focus on T cells and myeloid subsets known to play pivotal roles in the pathogenesis of EAE; (ii) identify, through an unsupervised approach, immune cell subsets that may serve as undescribed players in EAE. This analysis could provide insights into the molecular mechanisms of action specific to each KO mouse group.

Conventional flow cytometric analysis in the spleen revealed that the most affected subset within the pool of CD8^+^ T cells was the T_CM_ cells in both cohorts of KO, with a reduction trend in their absolute counts. A lower number of these cells in the spleen may reflect their migration to CNS, where they intensify inflammation and contribute to the destruction of myelin sheets. This phenomenon may be a contributing factor to the severe EAE observed in ICOSL-KO mice [47]. On the other hand, the CD4^+^ compartment was seemingly unaltered, at least at the level of total CD4^+^ cells. Nevertheless, the fluctuations regarding the absolute counts of T_EM_ cells in both KO groups resulted in a significant increase in ICOSL-KO and C57BL/6J mice in comparison with ICOS-KO mice. The major characteristic of CD4^+^ T_CM_ cells is their homing to secondary lymphoid organs, whereas T_EM_ cells are mainly located in nonlymphoid tissues and acquire effector functions, such as cytokine production and killing, more rapidly than T_CM_ cells [48]. However, both T cell subsets are present in the blood and spleen [26].

The obtained results revealed the reduction in the CD4^+^ T_EM_ cells in the spleen of ICOS-KO mice, which was not surprising since the available literature findings have already indicated that ICOS is an important factor specifically for the generation or survival of T_EM_ cells [49,50,51]. Burmeister et al. have shown that ICOS critically controls the pool size of T_EM_ and Tregs (Foxp3^+^) in the steady state, as well as in Ag-specific immune reactions, by regulating the survival of T cells [49].

In addition, Mahajan et al. examined endogenous CD4^+^ memory T cells in ICOS-KO mice by employing MHC class II tetramers. Their analysis unveiled regular primary clonal expansion, the generation of memory clones, and a prolonged survival (up to 10 weeks) of memory cells. However, these memory cells did not expand upon reactivation *in vitro*. These findings suggest that ICOS likely plays a role in bolstering secondary, memory, and effector T-cell responses, potentially by affecting cell survival [52]. Nevertheless, this still leaves open the question of the EAE severity that we observed in ICOS-KO mice.

In an MOG-induced EAE model, recovery is mediated by the expansion of Tregs [53]. It is well known that Tregs depletion results in increased incidence and accelerated disease onset in EAE model [53]. The absence of ICOS, under steady-state conditions, was shown to lead to a reduced number of Foxp3^+^ Tregs [54]. In our experiments, we found that both ICOSL-KO and ICOS-KO mice significantly reduced the recovery of symptoms in the remission phase, accompanied by a decline in CD4^+^Foxp3^+^ T cells, in comparison to C57BL/6J mice; this reduction is more prominent in ICOSL-KO mice. Our results coincide with several reports showing that the reduction or ablation of Tregs exacerbates EAE [55,56]. Interestingly, we found that Tregs in both ICOS-KO and ICOSL-KO mice co-express Foxp3 and Helios, identifying the activated phenotype [57]. Indeed, Helios, by directly binding to the promoter of Foxp3, increases its levels, contributing to the stability of Tregs [58]. ICOSL-KO mice, but not ICOS-KO mice, showed a trend in the reduction of activated Tregs, which could explain the severity of EAE seen in ICOSL-KO mice. However, Foxp3^+^Helios^+^ Tregs did not protect ICOS-KO mice from EAE. This is in line with a study from Golding and colleagues that showed that Helios-expressing Foxp3^+^ Tregs in systemic lupus erythematosus patients did not produce effector cytokines, and they still failed to fully protect the host from intense self-reactive B- and T-cell responses [59].

ICOSL is a costimulatory molecule expressed by APCs such as macrophages, DCs, and B cells. Our data showed that its deficiency results in an increase in the absolute numbers of macrophages and DCs during EAE remission. The number of macrophages is reported to be decreased during the resolution and recovery phases of EAE [60], as we observed in C57BL/6J mice, in which EAE was less severe compared to ICOSL-KO mice. Activated DCs could prime autoreactive T cells in secondary lymphatic organs, which then can become mobile, pass the endothelial barrier, and migrate to CNS, leading to inflammation [61,62]. Monocytic and granulocytic MDSC resulted in an increase in ICOSL-KO mice. One reason for this could be that, since, in these mice, OPN is unable to bind to ICOSL, it is instead free to bind to integrins, facilitating the expansion of MDSCs, and therefore leading to a more severe EAE. In line with this, it has been shown that depletion of MDSCs resulted in a marked reduction in EAE severity in the lymphoid tissues and spinal cords [63].

Conventional supervised flow cytometry analyses are limited to pre-defined cell populations and do not exploit the full potential of the data, especially when the panel includes several markers (>8). The increasing capacities of novel flow cytometers allows for the application of multiparametric flow cytometry and an unbiased analysis that uses all the included markers simultaneously to assess the similarity between cells. Clustering is an unsupervised learning technique since it categorizes unlabeled instances into meaningful groups using their similar properties. The advantage of using unsupervised clustering analysis is that it opens up the possibility to partition the data more finely by facilitating the finding of novel and unexpected populations that could be relevant in that specific disease. In our study, through unsupervised analysis, we identified a total of 10 clusters that exhibited differential expression between ICOS-KO and ICOSL-KO mice, as well as each KO group, compared to C57BL/6J ones.

ICOSL-KO mice showed a reduction in the macrophage subset (cluster 2), short-lived CD8^+^ T_EM_ cells (cluster 3), short-lived CD4^+^ T cells (cluster 4), and CD8^+^ Tregs (cluster 14) in comparison with ICOS-KO, suggesting that other mechanisms (i.e., the binding of ICOSL to OPN or ανβ_3_) may possibly support their expansion or maintenance. OPN is involved in the modulation of the generation of CD8^+^ memory T cells [64] and is still upregulated within the remission phase [65]. However, ICOSL binding to its new partners could inhibit the expansion of the monocytic-DC (cluster 5), resulting in a decrease in ICOS-KO mice: cells expressing the Ly6C marker migrate into the CNS and further differentiate into APCs during disease progression by promoting inflammation and tissue damage during EAE [66].

Moreover, we identified a novel cluster that encompasses Helios and Ly6C markers, which was reduced in ICOSL-KO mice when compared to the C57BL/6J mice cohort. This particular cluster is indicative of peripheral Tregs. The presence of Ly6C on Tregs is associated with a lower activation, proliferation, and differentiation status, as well as functional incompetence [35]. In summary, the decreased expression of this cluster in ICOSL-KO mice suggests potential alterations in the peripheral Treg population, pointing towards a nuanced impact on their activation and functional characteristics in the absence of ICOSL signaling. Conversely, we can speculate that the ICOS/ICOSL interaction could be responsible for long-lived CD4^+^ T_EM_ cells (cluster 18) in C57BL/6J mice, where these cells are at their highest levels compared to the KO mice. This cell population has already been described by MacLeod et al. [67,68,69].

Even though the supervised analysis did not reveal any significant difference in the myeloid compartment of the three examined groups of mice, we found that all cell clusters expressed the CD44 molecule. CD44 is a cell-surface glycoprotein involved in various cell-to-cell interactions such as cell adhesion and migration. Notably, CD44 is not exclusive to T and B cells, but is also expressed in natural killer cells, macrophages, DCs, and other cells [70].

The newly identified clusters in myeloid compartments exhibited differential modulation in both ICOS-KO and ICOSL-KO mice compared to C57BL/6J mice. Specifically, in ICOS-KO mice, we observed a significant increase in a cell subset expressing F4/80 but lacking CD11b, as well as a decrease in the cell population expressing CD11b and Ly6C markers but lacking the F4/80 marker. The former subset has been described by Tacke et al. as a novel tissue-resident macrophage population expressing only F4/80 in the thymus, where it engages in phagocytic activity by engulfing apoptotic thymocytes [71].

Lastly, ICOS-KO mice showed a significant increase in a cell population co-expressing CD11b, CD11c, and CD4 markers: this subset has been described as subpopulation of tolerogenic DCs, playing an important role in intravenous tolerance-induced EAE suppression [38].

In summary, although an unsupervised analysis could not fully explain the exacerbation of EAE seen in ICOSL and ICOS-KO mice, it provided valuable insights into the modulation of specific immune cell subsets in both KO mice.

Our study has two minor limitations. The first depends on the lack of non-MOG injected mice. Indeed, the EAE model that was used is driven by an encephalitogenic emulsion composed of the MOG antigen dissolved in CFA, supplemented with *M. tuberculosis* (4 mg/mL) as adjuvants. This is a well-established method to trigger a specific autoimmune response, mimicking certain aspects of MS in a controlled experimental setting [72,73,74], but the ability of CFA to induce a severe immune response against non-CNS antigens on its own [75], or to skew the immune response towards Th1-driven response [76], raised several concerns. As of today, most of the concerns raised regarding CFA refer to the rat model of EAE [77]. Efforts have been made to remove CFA from the immunization regimen in EAE studies. However, replacing CFA with an incomplete Freund’s adjuvant, which lacks *Mycobacterium*, which is believed to be accountable for the mentioned effects, has proven impractical in mice, as it induced tolerance instead [74]. Even though it is highly recommended to have no MOG_35–55_, Hasselmann et al. have shown that mice without MOG_35–55_ do not develop clinical symptoms of EAE [78].

The second limitation regards the lack of data investigating immune cell profile infiltrate in brains or spinal cords. Thus, we cannot rule out that the immune cell distribution in the spleen may be different to that in target organs. Murphy et al. have examined the brain infiltrates of CD4^+^ T cells secreting IL-17 and IFN-γ at 7, 10, 14, and 21 days post immunization, and demonstrated that the percentage of infiltrated CD4^+^ T cells secreting IL-17 in the brain was significantly diminished 21 days post-immunization, underscoring the resolution of the inflammatory process with the persistence of a memory response moving to the secondary lymphatic tissues, such as the spleen [79].

## 4. Materials and Methods

### 4.1. Induction and Clinical Evaluation of Experimental Autoimmune Encephalomyelitis (EAE)

Eight-to-ten-week-old adult female mice of the following three standard inbred strains were used: C57BL/6J, B6.129S6(Cg)-Spp1tm1Blh/J, also known as ICOS-KO, and B6.129P2-Icosltm1Mak/J, also known as ICOSL-KO (The Jackson Laboratory, Harbor, ME, USA). All mice were bred in our animal facility. EAE was induced by subcutaneous (s.c.) injection of 200 µg of MOG_35-55_ in CFA at the final concentration of 4 mg/mL, as previously described [80]. Pertussis toxin (500 ng) was injected intraperitoneally (i.p.) on days 0 and 2. Mice were observed from day 0, and the score of EAE clinical symptoms was recorded for 32 days according to the following classification: 0, no clinical signs; 1, loss of tail tone; 2, wobbly gait; 3, hind limb paralysis; 4, hind and fore limb paralysis; and 5, death. The animals were housed in a pathogen-free environment within the animal facility of Università del Piemonte Orientale. They had unrestricted access to rodent chow and water in their home cages, and the ambient temperature was consistently maintained at 21 ± 1 °C. All experimental procedures were carried out during the light phase of a 12:12 h light:dark cycle. At 7 days post-immunization, we observed two deceased mice. At the end point (day 32), mice were sacrificed by cervical dislocation and spleen were harvested for flow cytometry and enzyme-linked immunosorbent assay (ELISA), respectively.

### 4.2. MOG_35–55_-Induced Cytokine Release

Spleens were collected during the remission phase on day 32. Single-cell suspensions were obtained by passing through a 100 µM cell strainer (Becton and Dickinson, San José, CA, USA). Splenocytes (2 × 10^5^) were then cultured in complete RPMI 1640 media (GIBCO, Thermofisher, Waltham, MA, USA) containing 10% fetal bovine serum, in the absence or presence of MOG_35–55_ peptide (Espikem, Prato, Italy) (10 µg/mL). Supernatants were collected after 72 h of culture and kept at −20 °C until use. IFN-γ, IL-17A and IL-10 cytokines were quantified by ELISA MAX™ Standard Set Mouse according to the manufacturer’s instructions (Biolegend, San Diego, CA, USA).

### 4.3. Analysis of IL-17 Expression in Brain and Spinal Cord Tissues

Harvested brains and spinal cords were weighed, flash-frozen in liquid nitrogen, and stored at −80 °C until processing. Lysates of soluble proteins were prepared according to Bennett et al.’s protocol [81]. In brief, brain tissue was homogenized using a mortar and pestle and immediately transferred into 1.5 mL microcentrifuge tubes in RIPA buffer (PBS 1x, 1% nonylphenoxy polyethoxy ethanol, 0.5% sodium deoxycholate, 0.1% sodium doedecyl sulfate). Samples were centrifuged twice at 12,000 rpm for 20 min at 4 °C. The supernatant representing the total cell lysate was used in the ELISA protocol. Protein concentration was determined by Bradford Assay (Thermo Fisher Scientific, Waltham, MA, USA). A total of 5 (brain) and 20 (spinal cord) μg of protein lysate were used per well. IL-17A was quantified by ELISA according to the manufacturer’s instructions (R&D Systems, Minneapolis, MN, USA).

### 4.4. Flow Cytometry

Splenocytes were obtained by smashing the spleens through a cell strainer and red blood cells were lysed by osmotic shock. A total of 1 × 10^6^ of splenocytes were used for FACS staining. To discriminate live cells, 200 μL of Fixable Viability Dye 780 (Becton and Dickinson, San José, CA, USA) was added to each tube and cells were incubated for 15 min at 4 °C. Following this, cells were washed in 1 mL of PBS EDTA 2 mM and centrifuged for 5 min at 1500 rpm. After the centrifugation step, Fc receptor blocking was performed by incubating splenocytes with 100 μL of Anti-Mo CD16/CD32 (clone: 93) (eBioscience, Waltham, MA, USA) for 15 min at 4 °C, and then cells were washed as described above. We designed a panel consisting of 14 markers, which allowed for the phenotyping of lymphocytes (T cells and their respective subsets) and myeloid cells (macrophages, monocytic–myeloid-derived suppressor cells (MDSC)), granulocytic-MDSC and DCs. Splenocytes were subjected to surface staining using the following antibodies: mouse anti-CD3 BB700 monoclonal antibody (mAb) (clone: 145-2C11); anti-CD25 BB515 mAb (clone: C61); anti-Ly6C BV450 mAb (clone: AL-21); anti-CD45 BUV395 mAb (clone: 30-F11); anti-CD8 BV605 mAb (clone: 53-6.7); anti-CD4 BUV496 mAb (clone: GK1.5); anti-CD44 BV650 mAb (clone: IM7); anti-CD62L BUV737 mAb (clone: MEL-14); anti-CD11b BUV661 mAb (clone: M1/70); anti-CD11c BV480 mAb (clone: HL3); anti-Ly6G BUV563 mAb (clone: 1A8); and anti-F4/80 APC-R700 mAb (clone: T45-2342) for 30 min at 4 °C. All antibodies were purchased from BD Biosciences. Fluorescence minus one (FMOs) served as the control for the gating strategy. After washing, splenocytes were fixed and permeabilized using 100 μL of fixation and permeabilization solution (Cytofix/Cytoperm BD) and incubated overnight at 4 °C. On the next day, splenocytes were washed with 1 mL of permeabilization buffer and centrifuged. For intracellular staining, cells were incubated with anti-HELIOS PE mAb (clone: 22F6) and anti-Foxp3 Alexa Fluor 647 mAb (clone: MF23) and incubated for 20 min at 4 °C, after which the cells were washed and resuspended in 200 μL of PBS EDTA 2 mM. All the samples were acquired using FACSymphony^TM^ A5 (Becton and Dickinson, San José, CA, USA) flow cytometer and data were analyzed using FACSDIVA software (Version 9.1, Becton and Dickinson, San José, CA, USA ). Absolute cell numbers were calculated by multiplying the percentage of cells within designated gates by the total splenocytes count, obtained through trypan blue exclusion [82]. The entire dataset was analyzed following the conventional supervised gating strategy and in parallel with unsupervised analysis to exploit the full potential of the data generated by the co-expression of several markers, allowing for the identification of new clusters, which were unknown before running the algorithm. First, we used the UMAP algorithm for the multidimensionality reduction (FlowJo Version 10 software), and subsequently we identified different immunological clusters (X-shift algorithm). Then, a heatmap was generated and used to decipher all antigens of each cluster; the percentage of each cluster was compared with C57BL/6J, ICOSL-KO, and ICOS-KO mice. The gating strategy and FMOs are shown in Supplementary Figures S1–S3.

### 4.5. Statistical Analysis

EAE scores, flow cytometry and cytokine data were analyzed using ANOVA or Kruskal–Wallis test with post hoc correction, according to the sample’s normality, calculated using D’Agostino–Pearson test. A *p* value below 0.05 was considered statistically significant. Statistical analyses were conducted using GraphPad Instat software (Prism 8 version 8.4.3) (San Diego, CA, USA).

## 5. Conclusions

In conclusion, we investigated the impact of ICOSL and ICOS deficiencies on MOG_35-55_-induced EAE and associated immune cell dynamics in the resolution phase, using ICOSL-KO and ICOS-KO mice and comparing the results to C57BL/6J mice.

ICOSL deficiency led to the exacerbation of EAE compared to C57BL/6J mice, an overlapping clinical course with that of ICOS-KO mice, and a reduced recovery rate compared to C57BL/6J mice. ICOS and its ligand are deeply implicated in the differentiation and maintenance of Th17 cells secreting IL-17A. MOG-restimulated splenocytes from ICOS-KO and ICOSL-KO mice displayed reduced expression levels of IL-17A, which is also found in the spinal cord. Conventional flow cytometric analysis revealed a decrease in central memory CD8^+^ T cells in ICOSL-KO mice, while CD4^+^ T_EM_ cell counts were increased. Lastly, in addition to the absolute number of Tregs being lower in ICOSL-KO, they were also found to be less activated.

Unsupervised analysis identified 10 clusters with a differential expression between ICOS-KO and ICOSL-KO mice and C57BL/6J mice. ICOSL-KO mice showed a reduction in macrophage subsets, short-lived CD8^+^ T _EM_ cells, short-lived CD4^+^ T cells, and CD8^+^ Tregs compared to ICOS-KO. In addition, modulation in myeloid cell subsets, including changes in F4/80^+^CD11b^-^ (cluster 2) and CD11b^+^ Ly6C^+^ CD44^+^ (cluster 5) populations, were recorded.

In summary, the study provides comprehensive insights into the complex interplay between ICOS and ICOSL in the context of EAE. We believe our findings help to shed light on their roles in immune cell dynamics and the involvement of other ICOSL-driven mechanisms independent of ICOS, thus opening avenues for research into alternative pathways and potential therapeutic targets. Strategies aimed at modulating these pathways could be investigated for their effectiveness in controlling autoimmune responses and mitigating disease severity.

## Figures and Tables

**Table 1:** Basic phenotypes and affiliations of the defined clusters. The absence of ICOSL affects predominantly cells from myeloid compartment. Unsupervised analysis revealed significant decrease in macrophages (cluster 2) in ICOSL-KO in comparison to ICOS-KO group and a significant increase in monocytic-DC cells (cluster 5), in inflammatory monocytes (cluster 10) and naïve CD8^+^ cells (cluster 16) when compared to C57BL/6J mice. In addition, the absence of ICOSL had a negative effect on the long-lived effector memory of CD4^+^ T cells (decreased in cluster 18) and activated peripheral Tregs (cluster 8) when compared to wt mice. In comparison to ICOS-KO, the significant decrease in short-lived effector cells, both CD4 and CD8 (cluster 3 and 4, respectively), as well as immunosuppressive Tregs (cluster 14), was recorded.

Cluster	Markers (Phenotype)	Reference	ICOS-KO vs. C57BL/6J	ICOSL-KO vs. C57BL/6J	ICOS-KO vs. ICOSL-KO
2	F4/80^+^ CD44^+^ (macrophages)	[31]	x	x	↑
3	CD8^+^ CD44^+^ CD62L^−^ Ly6C^+^ (short-lived CD8^+^ T_EM_ cells)	[32]	↑	x	↑
4	CD4^+^ CD44^+^ Ly6C^+^ (short-lived effector CD4^+^T cells)	[33]	↑	x	↑
5	CD11b^+^ Ly6C^+^ CD44^+^ (monocytic-DC cells)	[34]	↓	x	↓
8	Ly6C^+^ Helios^+^ (peripheral Ly6C^+^ Tregs)	[35]	x	↓	x
10	CD11b^+^ Ly6C^+^ CD62L^+^ CD44^+^ (inflammatory monocytes)	[36]	x	↑	x
14	CD11c^+^ CD8^+^ CD44^+^ (CD8^+^ immunosuppressive Tregs)	[37]	x	x	↑
15	CD4^+^, CD11b^+^, CD11c^+^, CD44^+^ (tolerogenic DCs)	[38]	x	↑	x
16	CD3^+^ CD8^+^ Ly6C^+^ CD62L^+^ (naïve CD8^+^ T cells)	[39]	↓	x	x
18	CD3^+^ CD4^+^ CD44^+^ Ly6C^−^ (long-lived CD4^+^ T_EM_ cells)	[35]	↓	↓	x

## Data Availability

Data supporting the conclusions are presented in the manuscript. The datasets used and/or analyzed during the current study are available from the corresponding author on reasonable request.

## Supplementary Materials

The following supporting information can be downloaded at: https://www.mdpi.com/article/10.3390/ijms25052509/s1.

## References

[1] Dong C., Juedes A.E., Temann U.A., Shresta S., Allison J.P., Ruddle N.H., Flavell R.A. (2001). ICOS co-stimulatory receptor is essential for T-cell activation and function. Nature.

[2] Hedl M., Lahiri A., Ning K., Cho J.H., Abraham C. (2014). Pattern recognition receptor signaling in human dendritic cells is enhanced by ICOS ligand and modulated by the Crohn’s disease ICOSLG risk allele. Immunity.

[3] Swallow M.M., Wallin J.J., Sha W.C. (1999). B7h, a novel costimulatory homolog of B7.1 and B7.2, is induced by TNFalpha. Immunity.

[4] Galicia G., Kasran A., Uyttenhove C., De Swert K., Van Snick J., Ceuppens J.L. (2009). ICOS deficiency results in exacerbated IL-17 mediated experimental autoimmune encephalomyelitis. J. Clin. Immunol..

[5] Rottman J.B., Smith T., Tonra J.R., Ganley K., Bloom T., Silva R., Pierce B., Gutierrez-Ramos J.C., Ozkaynak E., Coyle A.J. (2001). The costimulatory molecule ICOS plays an important role in the immunopathogenesis of EAE. Nat. Immunol..

[6] Sporici R.A., Beswick R.L., von Allmen C., Rumbley C.A., Hayden-Ledbetter M., Ledbetter J.A., Perrin P.J. (2001). ICOS ligand costimulation is required for T-cell encephalitogenicity. Clin. Immunol..

[7] Dianzani C., Minelli R., Gigliotti C.L., Occhipinti S., Giovarelli M., Conti L., Boggio E., Shivakumar Y., Baldanzi G., Malacarne V. (2014). B7h triggering inhibits the migration of tumor cell lines. J. Immunol..

[8] Dianzani C., Minelli R., Mesturini R., Chiocchetti A., Barrera G., Boscolo S., Sarasso C., Gigliotti C.L., Sblattero D., Yagi J. (2010). B7h triggering inhibits umbilical vascular endothelial cell adhesiveness to tumor cell lines and polymorphonuclear cells. J. Immunol..

[9] Gigliotti C.L., Boggio E., Clemente N., Shivakumar Y., Toth E., Sblattero D., D’Amelio P., Isaia G.C., Dianzani C., Yagi J. (2016). ICOS-Ligand Triggering Impairs Osteoclast Differentiation and Function In Vitro and In Vivo. J. Immunol..

[10] Occhipinti S., Dianzani C., Chiocchetti A., Boggio E., Clemente N., Gigliotti C.L., Soluri M.F., Minelli R., Fantozzi R., Yagi J. (2013). Triggering of B7h by the ICOS modulates maturation and migration of monocyte-derived dendritic cells. J. Immunol..

[11] Raineri D., Dianzani C., Cappellano G., Maione F., Baldanzi G., Iacobucci I., Clemente N., Baldone G., Boggio E., Gigliotti C.L. (2020). Osteopontin binds ICOSL promoting tumor metastasis. Commun. Biol..

[12] Koh K.H., Cao Y., Mangos S., Tardi N.J., Dande R.R., Lee H.W., Samelko B., Altintas M.M., Schmitz V.P., Lee H. (2019). Nonimmune cell-derived ICOS ligand functions as a renoprotective αvβ3 integrin-selective antagonist. J. Clin. Invest..

[13] Raineri D., Cappellano G., Vilardo B., Maione F., Clemente N., Canciani E., Boggio E., Gigliotti C.L., Monge C., Dianzani C. (2021). Inducible T-Cell Costimulator Ligand Plays a Dual Role in Melanoma Metastasis upon Binding to Osteopontin or Inducible T-Cell Costimulator. Biomedicines.

[14] Horton M.A. (1997). The alpha v beta 3 integrin “vitronectin receptor”. Int. J. Biochem. Cell Biol..

[15] Hauser S.L., Oksenberg J.R. (2006). The neurobiology of multiple sclerosis: Genes, inflammation, and neurodegeneration. Neuron.

[16] Goverman J. (2009). Autoimmune T cell responses in the central nervous system. Nat. Rev. Immunol..

[17] Lassmann H., Suchanek G., Ozawa K. (1994). Histopathology and the blood-cerebrospinal fluid barrier in multiple sclerosis. Ann. Neurol..

[18] Lucchinetti C., Brück W., Parisi J., Scheithauer B., Rodriguez M., Lassmann H. (2000). Heterogeneity of multiple sclerosis lesions: Implications for the pathogenesis of demyelination. Ann. Neurol..

[19] Bauquet A.T., Jin H., Paterson A.M., Mitsdoerffer M., Ho I.C., Sharpe A.H., Kuchroo V.K. (2009). The costimulatory molecule ICOS regulates the expression of c-Maf and IL-21 in the development of follicular T helper cells and TH-17 cells. Nat. Immunol..

[20] Mesturini R., Gigliotti C.L., Orilieri E., Cappellano G., Soluri M.F., Boggio E., Woldetsadik A., Dianzani C., Sblattero D., Chiocchetti A. (2013). Differential induction of IL-17, IL-10, and IL-9 in human T helper cells by B7h and B7.1. Cytokine.

[21] Mesturini R., Nicola S., Chiocchetti A., Bernardone I.S., Castelli L., Bensi T., Ferretti M., Comi C., Dong C., Rojo J.M. (2006). ICOS cooperates with CD28, IL-2, and IFN-gamma and modulates activation of human naïve CD4+ T cells. Eur. J. Immunol..

[22] Yong P.F., Salzer U., Grimbacher B. (2009). The role of costimulation in antibody deficiencies: ICOS and common variable immunodeficiency. Immunol. Rev..

[23] Nurieva R.I. (2005). Regulation of immune and autoimmune responses by ICOS-B7h interaction. Clin. Immunol..

[24] Scott B.G., Yang H., Tüzün E., Dong C., Flavell R.A., Christadoss P. (2004). ICOS is essential for the development of experimental autoimmune myasthenia gravis. J. Neuroimmunol..

[25] Rojo J.M., Pini E., Ojeda G., Bello R., Dong C., Flavell R.A., Dianzani U., Portolés P. (2008). CD4+ICOS+ T lymphocytes inhibit T cell activation ‘in vitro’ and attenuate autoimmune encephalitis ‘in vivo’. Int. Immunol..

[26] Elyaman W., Kivisäkk P., Reddy J., Chitnis T., Raddassi K., Imitola J., Bradshaw E., Kuchroo V.K., Yagita H., Sayegh M.H. (2008). Distinct functions of autoreactive memory and effector CD4+ T cells in experimental autoimmune encephalomyelitis. Am. J. Pathol..

[27] Hensley-McBain T., Heit A., De Rosa S.C., McElrath M.J., Andersen-Nissen E. (2014). Optimization of a whole blood phenotyping assay for enumeration of peripheral blood leukocyte populations in multicenter clinical trials. J. Immunol. Methods.

[28] Sugita K., Hanakawa S., Honda T., Kondoh G., Miyachi Y., Kabashima K., Nomura T. (2015). Generation of Helios reporter mice and an evaluation of the suppressive capacity of Helios(+) regulatory T cells in vitro. Exp. Dermatol..

[29] Armstrong G., Martino C., Rahman G., Gonzalez A., Vázquez-Baeza Y., Mishne G., Knight R. (2021). Uniform Manifold Approximation and Projection (UMAP) Reveals Composite Patterns and Resolves Visualization Artifacts in Microbiome Data. mSystems.

[30] Samusik N., Good Z., Spitzer M.H., Davis K.L., Nolan G.P. (2016). Automated mapping of phenotype space with single-cell data. Nat. Methods.

[31] Lee Y.H., Thacker R.I., Hall B.E., Kong R., Granneman J.G. (2014). Exploring the activated adipogenic niche: Interactions of macrophages and adipocyte progenitors. Cell Cycle.

[32] Samji T., Khanna K.M. (2017). Understanding memory CD8(+) T cells. Immunol. Lett..

[33] Marshall H.D., Chandele A., Jung Y.W., Meng H., Poholek A.C., Parish I.A., Rutishauser R., Cui W., Kleinstein S.H., Craft J. (2011). Differential expression of Ly6C and T-bet distinguish effector and memory Th1 CD4(+) cell properties during viral infection. Immunity.

[34] Plantinga M., Guilliams M., Vanheerswynghels M., Deswarte K., Branco-Madeira F., Toussaint W., Vanhoutte L., Neyt K., Killeen N., Malissen B. (2013). Conventional and monocyte-derived CD11b(+) dendritic cells initiate and maintain T helper 2 cell-mediated immunity to house dust mite allergen. Immunity.

[35] Lee J.Y., Kim J., Yi J., Kim D., Kim H.O., Han D., Sprent J., Lee Y.J., Surh C.D., Cho J.H. (2018). Phenotypic and Functional Changes of Peripheral Ly6C(+) T Regulatory Cells Driven by Conventional Effector T Cells. Front. Immunol..

[36] Xu H., Manivannan A., Crane I., Dawson R., Liversidge J. (2008). Critical but divergent roles for CD62L and CD44 in directing blood monocyte trafficking in vivo during inflammation. Blood.

[37] Vinay D.S., Kim C.H., Choi B.K., Kwon B.S. (2009). Origins and functional basis of regulatory CD11c+CD8+ T cells. Eur. J. Immunol..

[38] Wang L., Li Z., Ciric B., Safavi F., Zhang G.X., Rostami A. (2016). Selective depletion of CD11c(+) CD11b(+) dendritic cells partially abrogates tolerogenic effects of intravenous MOG in murine EAE. Eur. J. Immunol..

[39] Lee S.W., Lee G.W., Kim H.O., Cho J.H. (2023). Shaping Heterogeneity of Naive CD8(+) T Cell Pools. Immune Netw..

[40] Coyle A.J., Lehar S., Lloyd C., Tian J., Delaney T., Manning S., Nguyen T., Burwell T., Schneider H., Gonzalo J.A. (2000). The CD28-related molecule ICOS is required for effective T cell-dependent immune responses. Immunity.

[41] Hutloff A., Dittrich A.M., Beier K.C., Eljaschewitsch B., Kraft R., Anagnostopoulos I., Kroczek R.A. (1999). ICOS is an inducible T-cell co-stimulator structurally and functionally related to CD28. Nature.

[42] McAdam A.J., Chang T.T., Lumelsky A.E., Greenfield E.A., Boussiotis V.A., Duke-Cohan J.S., Chernova T., Malenkovich N., Jabs C., Kuchroo V.K. (2000). Mouse inducible costimulatory molecule (ICOS) expression is enhanced by CD28 costimulation and regulates differentiation of CD4+ T cells. J. Immunol..

[43] Tafuri A., Shahinian A., Bladt F., Yoshinaga S.K., Jordana M., Wakeham A., Boucher L.M., Bouchard D., Chan V.S., Duncan G. (2001). ICOS is essential for effective T-helper-cell responses. Nature.

[44] Paulos C.M., Carpenito C., Plesa G., Suhoski M.M., Varela-Rohena A., Golovina T.N., Carroll R.G., Riley J.L., June C.H. (2010). The inducible costimulator (ICOS) is critical for the development of human T(H)17 cells. Sci. Transl. Med..

[45] Quiroga M.F., Pasquinelli V., Martínez G.J., Jurado J.O., Zorrilla L.C., Musella R.M., Abbate E., Sieling P.A., García V.E. (2006). Inducible costimulator: A modulator of IFN-gamma production in human tuberculosis. J. Immunol..

[46] Park H., Li Z., Yang X.O., Chang S.H., Nurieva R., Wang Y.H., Wang Y., Hood L., Zhu Z., Tian Q. (2005). A distinct lineage of CD4 T cells regulates tissue inflammation by producing interleukin 17. Nat. Immunol..

[47] Ritzel R.M., Crapser J., Patel A.R., Verma R., Grenier J.M., Chauhan A., Jellison E.R., McCullough L.D. (2016). Age-Associated Resident Memory CD8 T Cells in the Central Nervous System Are Primed To Potentiate Inflammation after Ischemic Brain Injury. J. Immunol..

[48] Sallusto F., Lenig D., Förster R., Lipp M., Lanzavecchia A. (1999). Two subsets of memory T lymphocytes with distinct homing potentials and effector functions. Nature.

[49] Burmeister Y., Lischke T., Dahler A.C., Mages H.W., Lam K.P., Coyle A.J., Kroczek R.A., Hutloff A. (2008). ICOS controls the pool size of effector-memory and regulatory T cells. J. Immunol..

[50] Moore T.V., Clay B.S., Ferreira C.M., Williams J.W., Rogozinska M., Cannon J.L., Shilling R.A., Marzo A.L., Sperling A.I. (2011). Protective effector memory CD4 T cells depend on ICOS for survival. PLoS ONE.

[51] Simpson T.R., Quezada S.A., Allison J.P. (2010). Regulation of CD4 T cell activation and effector function by inducible costimulator (ICOS). Curr. Opin. Immunol..

[52] Mahajan S., Cervera A., MacLeod M., Fillatreau S., Perona-Wright G., Meek S., Smith A., MacDonald A., Gray D. (2007). The role of ICOS in the development of CD4 T cell help and the reactivation of memory T cells. Eur. J. Immunol..

[53] Koutrolos M., Berer K., Kawakami N., Wekerle H., Krishnamoorthy G. (2014). Treg cells mediate recovery from EAE by controlling effector T cell proliferation and motility in the CNS. Acta Neuropathol. Commun..

[54] Li D.Y., Xiong X.Z. (2020). ICOS(+) Tregs: A Functional Subset of Tregs in Immune Diseases. Front. Immunol..

[55] McGeachy M.J., Stephens L.A., Anderton S.M. (2005). Natural recovery and protection from autoimmune encephalomyelitis: Contribution of CD4+CD25+ regulatory cells within the central nervous system. J. Immunol..

[56] Montero E., Nussbaum G., Kaye J.F., Perez R., Lage A., Ben-Nun A., Cohen I.R. (2004). Regulation of experimental autoimmune encephalomyelitis by CD4+, CD25+ and CD8+ T cells: Analysis using depleting antibodies. J. Autoimmun..

[57] Akimova T., Beier U.H., Wang L., Levine M.H., Hancock W.W. (2011). Helios expression is a marker of T cell activation and proliferation. PLoS ONE.

[58] Getnet D., Grosso J.F., Goldberg M.V., Harris T.J., Yen H.R., Bruno T.C., Durham N.M., Hipkiss E.L., Pyle K.J., Wada S. (2010). A role for the transcription factor Helios in human CD4(+)CD25(+) regulatory T cells. Mol. Immunol..

[59] Golding A., Hasni S., Illei G., Shevach E.M. (2013). The percentage of FoxP3+Helios+ Treg cells correlates positively with disease activity in systemic lupus erythematosus. Arthritis Rheum..

[60] Ousman S.S., Kubes P. (2012). Immune surveillance in the central nervous system. Nat. Neurosci..

[61] Banchereau J., Steinman R.M. (1998). Dendritic cells and the control of immunity. Nature.

[62] Cella M., Döhring C., Samaridis J., Dessing M., Brockhaus M., Lanzavecchia A., Colonna M. (1997). A novel inhibitory receptor (ILT3) expressed on monocytes, macrophages, and dendritic cells involved in antigen processing. J. Exp. Med..

[63] Yi H., Guo C., Yu X., Zuo D., Wang X.Y. (2012). Mouse CD11b+Gr-1+ myeloid cells can promote Th17 cell differentiation and experimental autoimmune encephalomyelitis. J. Immunol..

[64] Morimoto J., Sato K., Nakayama Y., Kimura C., Kajino K., Matsui Y., Miyazaki T., Uede T. (2011). Osteopontin modulates the generation of memory CD8+ T cells during influenza virus infection. J. Immunol..

[65] Chabas D., Baranzini S.E., Mitchell D., Bernard C.C., Rittling S.R., Denhardt D.T., Sobel R.A., Lock C., Karpuj M., Pedotti R. (2001). The influence of the proinflammatory cytokine, osteopontin, on autoimmune demyelinating disease. Science.

[66] Monaghan K.L., Zheng W., Hu G., Wan E.C.K. (2019). Monocytes and Monocyte-Derived Antigen-Presenting Cells Have Distinct Gene Signatures in Experimental Model of Multiple Sclerosis. Front. Immunol..

[67] MacLeod M., Kwakkenbos M.J., Crawford A., Brown S., Stockinger B., Schepers K., Schumacher T., Gray D. (2006). CD4 memory T cells survive and proliferate but fail to differentiate in the absence of CD40. J. Exp. Med..

[68] MacLeod M.K., Clambey E.T., Kappler J.W., Marrack P. (2009). CD4 memory T cells: What are they and what can they do?. Semin. Immunol..

[69] MacLeod M.K., McKee A., Crawford F., White J., Kappler J., Marrack P. (2008). CD4 memory T cells divide poorly in response to antigen because of their cytokine profile. Proc. Natl. Acad. Sci. USA.

[70] Isacke C.M., Horton M.A. (2000). The Adhesion Molecule FactsBook.

[71] Tacke R., Hilgendorf I., Garner H., Waterborg C., Park K., Nowyhed H., Hanna R.N., Wu R., Swirski F.K., Geissmann F. (2015). The transcription factor NR4A1 is essential for the development of a novel macrophage subset in the thymus. Sci. Rep..

[72] Baxter A.G. (2007). The origin and application of experimental autoimmune encephalomyelitis. Nat. Rev. Immunol..

[73] Mangiardi M., Crawford D.K., Xia X., Du S., Simon-Freeman R., Voskuhl R.R., Tiwari-Woodruff S.K. (2011). An animal model of cortical and callosal pathology in multiple sclerosis. Brain Pathol..

[74] Mills K.H. (2011). TLR-dependent T cell activation in autoimmunity. Nat. Rev. Immunol..

[75] Namer I.J., Steibel J., Poulet P., Armspach J.P., Mohr M., Mauss Y., Chambron J. (1993). Blood-brain barrier breakdown in MBP-specific T cell induced experimental allergic encephalomyelitis. A quantitative in vivo MRI study. Brain.

[76] Billiau A., Matthys P. (2001). Modes of action of Freund’s adjuvants in experimental models of autoimmune diseases. J. Leukoc. Biol..

[77] Lazarević M., Djedovic N., Stanisavljević S., Dimitrijević M., Stegnjaić G., Krishnamoorthy G., Mostarica Stojković M., Miljković Đ., Jevtić B. (2021). Complete Freund’s adjuvant-free experimental autoimmune encephalomyelitis in Dark Agouti rats is a valuable tool for multiple sclerosis studies. J. Neuroimmunol..

[78] Hasselmann J.P.C., Karim H., Khalaj A.J., Ghosh S., Tiwari-Woodruff S.K. (2017). Consistent induction of chronic experimental autoimmune encephalomyelitis in C57BL/6 mice for the longitudinal study of pathology and repair. J. Neurosci. Methods.

[79] Murphy A.C., Lalor S.J., Lynch M.A., Mills K.H. (2010). Infiltration of Th1 and Th17 cells and activation of microglia in the CNS during the course of experimental autoimmune encephalomyelitis. Brain Behav. Immun..

[80] Cappellano G., Woldetsadik A.D., Orilieri E., Shivakumar Y., Rizzi M., Carniato F., Gigliotti C.L., Boggio E., Clemente N., Comi C. (2014). Subcutaneous inverse vaccination with PLGA particles loaded with a MOG peptide and IL-10 decreases the severity of experimental autoimmune encephalomyelitis. Vaccine.

[81] Bennett S.A., Roberts D.C. (2003). Analysis of protein expression in brain tissue by ELISA. Methods Mol. Med..

[82] Buszko M., Cardini B., Oberhuber R., Oberhuber L., Jakic B., Beierfuss A., Wick G., Cappellano G. (2017). Differential depletion of total T cells and regulatory T cells and prolonged allotransplant survival in CD3Ɛ humanized mice treated with polyclonal anti human thymocyte globulin. PLoS ONE.

