# Supplementary material for: Deep Flow Cytometry Unveils Distinct Immune Cell Subsets in Inducible T Cell Co-Stimulator Ligand (ICOSL)- and ICOS-Knockout Mice during Experimental Autoimmune Encephalomyelitis
Source: Int J Mol Sci. 2024 Feb 21;25(5):2509. doi: 10.3390/ijms25052509 (PMC10932052; doi:10.3390/ijms25052509)
Supplement: Supplementary file 1 [file ijms-25-02509-s001.zip › ijms-2824765-supplementary.pdf]

Supplementary Material

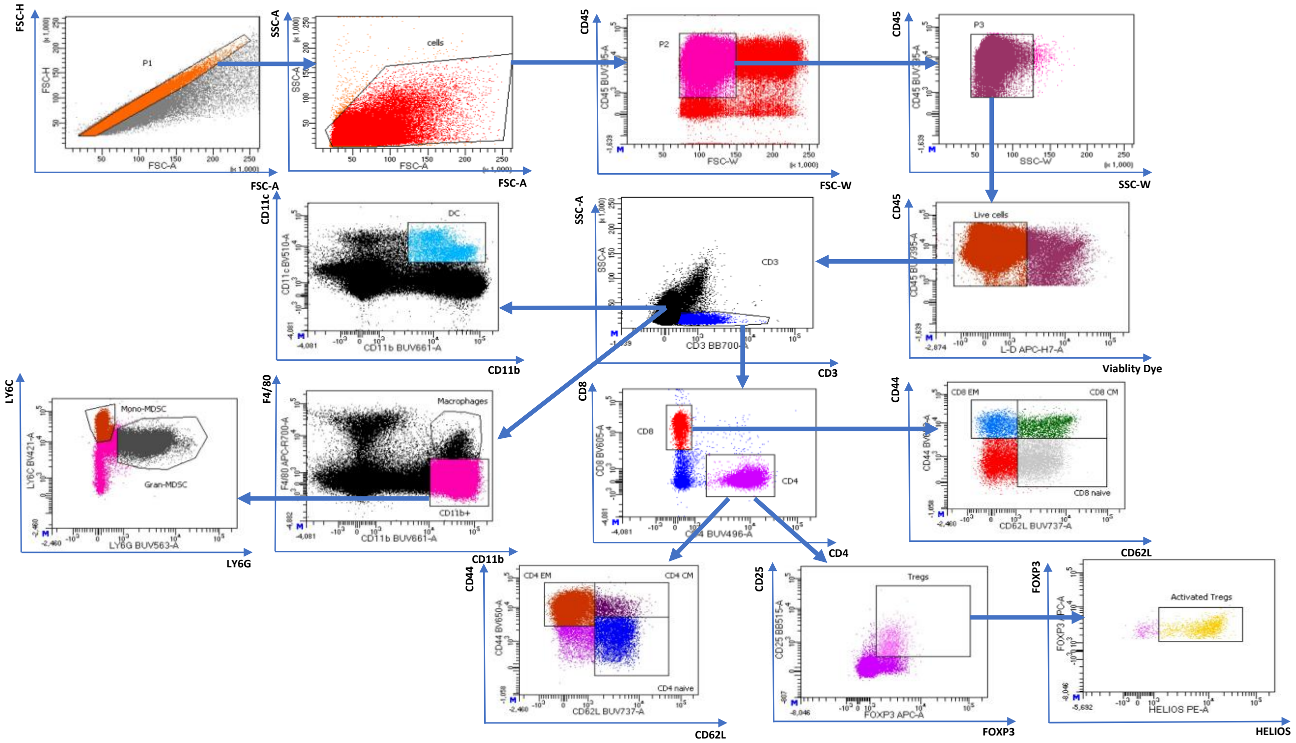

Supplementary Figure S1. Gating strategy. Arrows describe the hierarchical sequences of analysis. Singlets were selected from the FSC-A versus FSC-H dot plot and cells were gated based on SSC-A versus FSC-A. Then, CD45^{+}positive cells were discriminated accordingly to the width parameter (FSC-W, SSC-W) and dead cells were excluded with the viability dye. Based on CD45^{+} live cells, T cells were identified as CD3^{+}. CD4^{+} and CD8^{+} T cells were identified accordingly to the expression of their surface markers. In both CD4 and CD8 subsets, naïve cells were classified as CD44^{-} CD62L^{+}, central memory cells as CD44^{+} CD62L^{+} (CM) and effector memory cells (EM) as CD44^{+}CD62L^{-}. Tregs cells were classified as CD4^{+} CD25^{+} Foxp3^{+} and activated Tregs as Foxp3^{+}Helios^{+}. On CD3^{-} cells, dendritic cells (DC) were classified as CD11b^{+} CD11c^{+} cells and macrophages as CD11b^{+}F4/80^{+} cells. Finally, on CD11^{+} cells, monocytic myeloid suppressor cells (MDSC) and granulocytic -MDSC were

identified as Ly6C^{+}Ly6G^{-} and Ly6C^{-}Ly6G^{+}. Samples were acquired using FACSymphony^{TM} A5 (BD) flow cytometer and data were analyzed using DIVA software (BD).

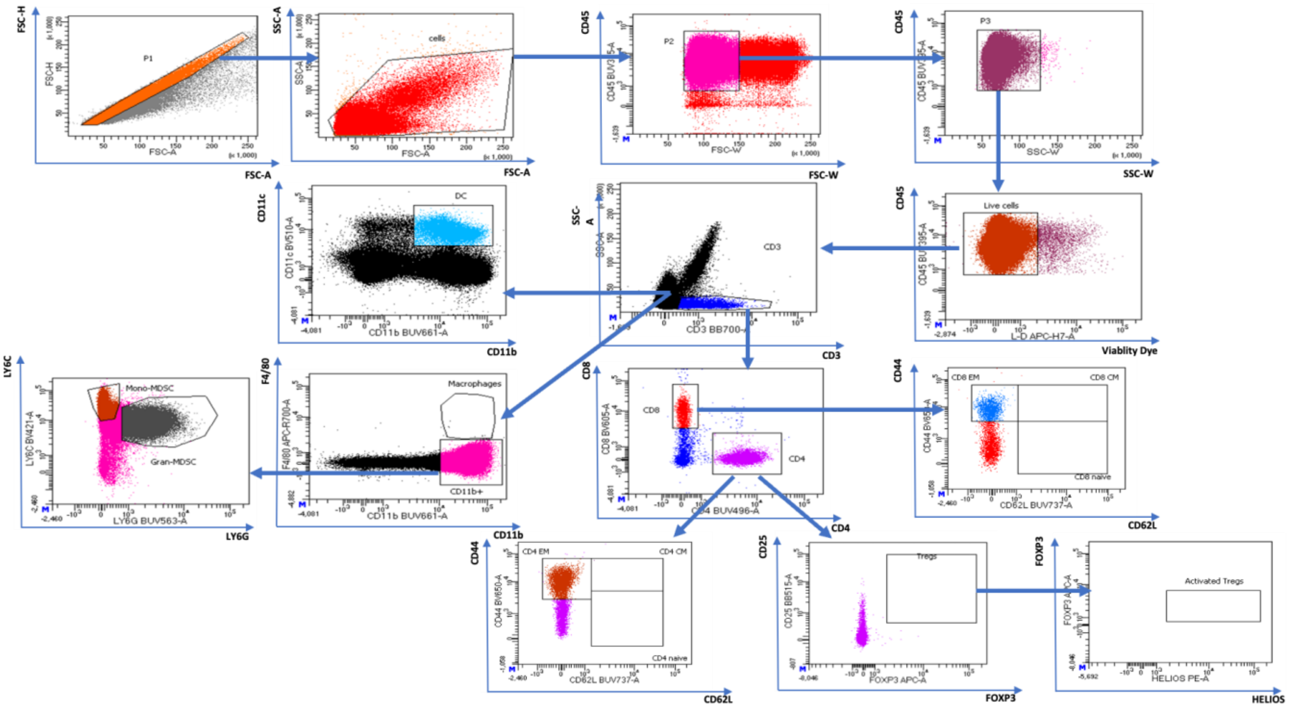

Supplementary Figure S2. Gating strategy, described in supplementary figure 1, showing the Fluorescence Minus One (FMO) controls for anti-F4/80, anti-CD62L and anti-Foxp3 mouse monoclonal antibodies.

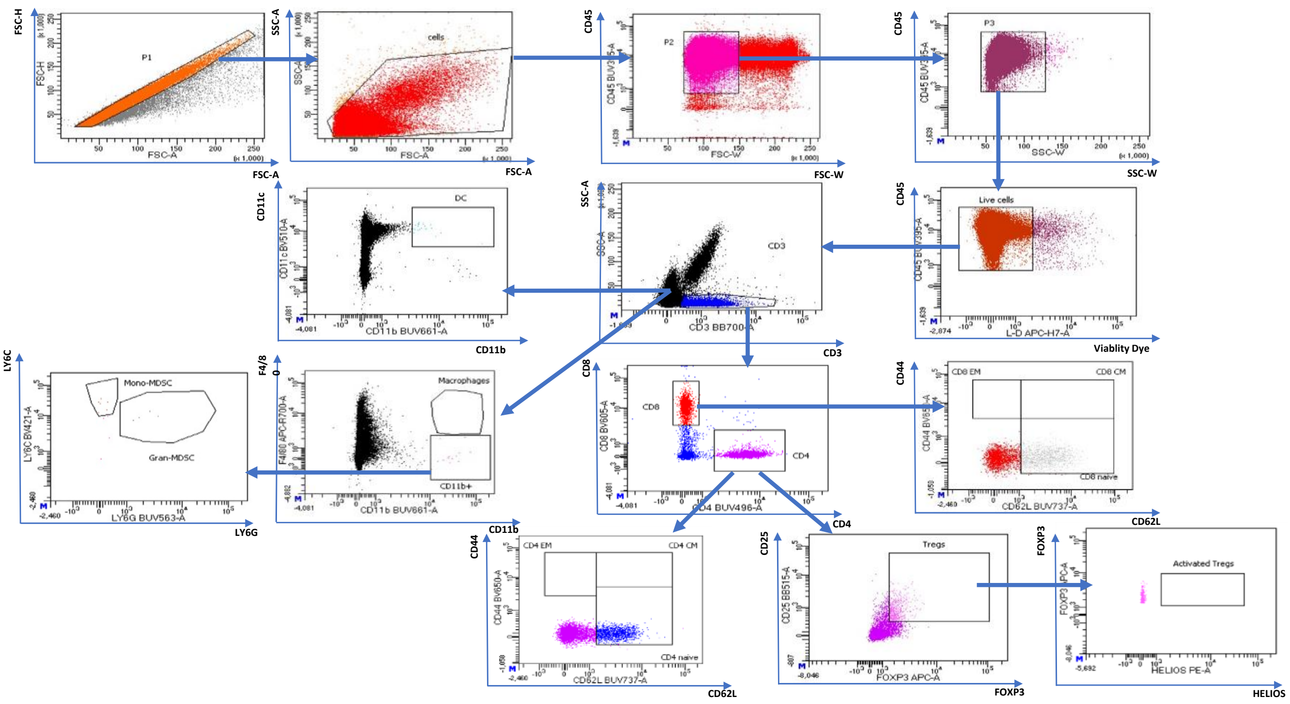

Supplementary Figure S3. Gating strategy, described in supplementary figure 1, showing the FMO controls for anti-CD11b, anti-CD44 and anti-Helios mouse monoclonal antibodies.
